# Supplementary material for: Mechanisms governing the pioneering and redistribution capabilities of the non-classical pioneer PU.1
Source: Nat Commun. 2020 Jan 21;11:402. doi: 10.1038/s41467-019-13960-2 (PMC6972792; doi:10.1038/s41467-019-13960-2)
Supplement: Supplementary file 7 — Source data [file 41467_2019_13960_MOESM7_ESM.zip › Source_Data/FigureS2/FigureS2B_MotifScanOutput/KmeansCluster10/homerResults/motif22.similar.html]

motif22

## Information for motif22

C
T
A
G
A
G
T
C
A
G
T
C
C
T
G
A
A
G
T
C
C
T
A
G
T
C
G
A
  
Reverse Opposite:  

A
G
C
T
A
G
T
C
A
C
T
G
A
G
C
T
A
C
T
G
A
C
T
G
A
G
T
C
  

|  |  |
| --- | --- |
| p-value: | 1e-30 |
| log p-value: | -6.932e+01 |
| Information Content per bp: | 1.860 |
| Number of Target Sequences with motif | 959.0 |
| Percentage of Target Sequences with motif | 25.84% |
| Number of Background Sequences with motif | 7906.6 |
| Percentage of Background Sequences with motif | 17.89% |
| Average Position of motif in Targets | 100.2 +/- 56.4bp |
| Average Position of motif in Background | 99.8 +/- 61.6bp |
| Strand Bias (log2 ratio + to - strand density) | 0.1 |
| Multiplicity (# of sites on avg that occur together) | 1.14 |
| Motif File: | file (matrix) reverse opposite |

### Similar de novo motifs found

|  |  |  |  |  |  |  |  |
| --- | --- | --- | --- | --- | --- | --- | --- |
| Rank | Match Score | Redundant Motif | P-value | log P-value | % of Targets | % of Background | Motif file |
